# Peritumoral brain zone oxygen extraction fraction is associated with tumor Ki-67 index in untreated glioblastoma

**DOI:** 10.1093/noajnl/vdag163

**Published:** 2026-06-18

**Authors:** Jonas Reis, Marco Öchsner, Antonia Neubauer, Chiara Adam, Louisa von Baumgarten, Darius Kalasauskas, Florian Ringel, Thomas Liebig, Nathalie L Albert, Patrick N Harter, Robert Forbrig

**Affiliations:** Institute of Neuroradiology, LMU University Hospital, LMU Munich, Germany; Institute of Neuroradiology, LMU University Hospital, LMU Munich, Germany; Center for Neuropathology and Prion Research, Faculty of Medicine, LMU Munich, Germany; Institute of Neuroradiology, LMU University Hospital, LMU Munich, Germany; Department of Neurology, LMU University Hospital, LMU Munich, Germany; Department of Neurosurgery, LMU University Hospital, LMU Munich, Germany; German Cancer Consortium (DKTK), Munich, Germany; Bavarian Cancer Research Center (BZKF), Munich, Germany; Department of Neurosurgery, LMU University Hospital, LMU Munich, Germany; Department of Neurosurgery, LMU University Hospital, LMU Munich, Germany; Institute of Neuroradiology, LMU University Hospital, LMU Munich, Germany; Department of Nuclear Medicine, LMU University Hospital, LMU Munich, Germany; German Cancer Consortium (DKTK), Munich, Germany; Bavarian Cancer Research Center (BZKF), Munich, Germany; Center for Neuropathology and Prion Research, Faculty of Medicine, LMU Munich, Germany; German Cancer Consortium (DKTK), Munich, Germany; Bavarian Cancer Research Center (BZKF), Munich, Germany; Institute of Neuroradiology, LMU University Hospital, LMU Munich, Germany

**Keywords:** contrast MRI, dynamic susceptibility, glioblastoma, Ki-67, oxygen extraction fraction, peritumoral brain zone

## Abstract

**Background:**

The non-contrast-enhancing peritumoral brain zone (PBZ) contributes to glioblastoma progression but remains incompletely characterized by structural MRI. We tested whether PBZ oxygen extraction fraction (OEF) derived from dynamic susceptibility contrast (DSC) MRI is associated with the Ki‑67 proliferation index at the patient level.

**Methods:**

In this retrospective analysis, we analyzed 80 adults with untreated *IDH*-wildtype glioblastoma with preoperative DSC and diffusion MRI. Mean values of paired regions of interest were extracted from non-contrast-enhancing T2/FLAIR-hyperintense PBZ, the contrast-enhancing tumor core, and contralateral normal-appearing white matter (NAWM). PBZ and CE to NAWM ratios for OEF, capillary transit time heterogeneity, cerebral metabolic rate of oxygen, cerebral blood volume, and apparent diffusion coefficient were computed after ComBat harmonization. These ratios were subsequently correlated with Ki-67 using Spearman’s rank correlation.

**Results:**

PBZ OEF demonstrated a positive correlation with Ki-67 (Spearman ρ = 0.27, *P* = .014). In contrast, other PBZ and all CE imaging ratios were not associated with Ki-67 after multiple testing. In regression models including CE OEF, PBZ OEF remained associated with Ki‑67 (*P* = .041) and improved model fit (ΔR^2^ = 0.094).

**Conclusions:**

Higher DSC-derived PBZ OEF ratio was modestly but significantly associated with higher tumor Ki-67 in untreated *IDH*-wildtype glioblastoma, suggesting that PBZ OEF captures physiologic variation in non-contrast-enhancing tissue beyond the contrast-enhancing tumor core. Given the lack of spatial co-registration between PBZ and pathology sampling, prospective validation with spatially matched histology and outcome endpoints is warranted.

Key Points

- Peritumoral brain zone (PBZ) OEF was positively associated with patient-level Ki-67 in untreated *IDH*-wildtype glioblastoma.
- Contrast-enhancing (CE) OEF was not associated with Ki‑67, and PBZ OEF added explanatory value beyond CE OEF in linear regression models.
- Other PBZ and CE metrics (CBV, CTH, CMRO_2_, ADC) were not significant after multiplicity control, supporting PBZ OEF as a candidate imaging biomarker that requires prospective validation with spatially matched tissue and outcome endpoints.

Importance of the Study

Most quantitative MRI biomarkers in glioblastoma focus on the contrast-enhancing (CE) core, whereas physiologic characterization of the non-contrast-enhancing peritumoral brain zone (PBZ), a compartment that is a frequent location of recurrence, remains limited. Using dynamic susceptibility contrast MRI with region-of-interest sampling, we quantified PBZ oxygen extraction fraction (OEF) normalized to contralateral normal-appearing white matter and tested its correlation with the proliferation index Ki-67 in untreated *IDH*-wildtype glioblastoma. Higher PBZ OEF was associated with higher Ki-67 and added explanatory value beyond CE OEF in secondary models. These findings support PBZ oxygen handling phenotyping as a candidate imaging biomarker linked to proliferation at the patient level. Because pathology sampling was not spatially co-registered, prospective evaluation with spatially matched biopsies and outcome endpoints are needed to establish reproducibility and incremental clinical utility.

The 2021 WHO CNS classification defines adult-type diffuse gliomas based on their genomic characteristics, but MRI remains essential for delineating tumor extent in vivo and for guiding surgery and radiotherapy.^1^^,^^2^ Structural MRI alone, however, has limited specificity for underlying tumor biology beyond the contrast‑enhancing (CE) rim.^3^^,^^4^ In glioblastoma, prognosis and treatment failure are frequently driven by non-contrast‑enhancing (non-CE) tissue surrounding the CE core, ie in the peritumoral brain zone (PBZ), which typically corresponds to T2/FLAIR-hyperintensity and reflects a heterogeneous mixture of infiltrating tumor, vasogenic edema, and a reactive microenvironment.^3^^,^^5-7^

Dynamic susceptibility‑contrast (DSC) MRI has evolved as a robust physiologic assay for brain tumors, with cerebral blood volume (CBV) and related metrics standardized by consensus protocols.^8^^,^^9^ While CBV primarily reflects vascular volume and perfusion capacity, oxygen‑metabolic imaging focuses on microvascular efficiency and tissue oxygenation. Capillary transit time heterogeneity (CTH) summarizes how uneven capillary transit times are within tissue. In flow-diffusion theory, greater heterogeneity increases the fraction of very short or functionally shunted paths (conceptually described as “malignant CTH”), such that higher flow may not improve oxygenation, whereas more homogeneous transit times support more efficient extraction.^10^^,^^11^ Advanced DSC capillary-function modeling allows joint, model-based estimation of CTH and oxygen extraction fraction (OEF) from the fitted transit-time distribution, offering physiologic information on microvascular oxygen handling that is complementary to CBV.^12^^,^^13^ Recent glioma studies have associated higher tumor OEF with more aggressive molecular characteristics and poorer outcomes.^14^^,^^15^ This motivates evaluating OEF and CTH specifically in the baseline assessment of PBZ, where conventional MRI provides limited biologic specificity.

Despite this rationale, to our knowledge, PBZ OEF and CTH have not been associated with global proliferative activity as indexed by Ki-67 in untreated *IDH*-wildtype glioblastoma. Because Ki‑67 is obtained from routine tumor sampling (typically from resected CE tumor), it represents a patient-level proliferative index rather than a spatially matched measure of PBZ biology. Accordingly, we primarily hypothesized that the NAWM-normalized PBZ OEF and CTH ratio is positively associated with Ki-67 at the patient level. Secondary analyses examined other PBZ metrics (cerebral metabolic rate of oxygen [CMRO_2_], CBV, and ADC) in relation to Ki-67 and evaluated CE metrics as a contextual comparison given the origin of Ki-67 sampling. Exploratory and supplemental analyses assessed potential effect heterogeneity across CTH and associations with selected molecular features.

## Methods

### Study Design and Cohort

We performed a single-center retrospective imaging-pathology study in patients with newly diagnosed untreated *IDH*-wildtype glioblastoma (WHO grade 4). Consecutive patients were identified from the institutional neuropathology database over a predefined accrual window (January 2021 to September 2024). Inclusion criteria were: (i) histopathologically confirmed *IDH*-wildtype glioblastoma according to the 2021 WHO CNS classification, (ii) preoperative MRI including gradient-echo (GRE) DSC perfusion, diffusion-weighted imaging (DWI), T2-/FLAIR-weighted imaging, and post-contrast T1-weighted imaging, and (iii) a reported Ki-67 index (%) from a tumor specimen. Exclusion criteria were severe motion or susceptibility artifacts, corrupted or incomplete DSC data, prior therapy, or absence of any T2/FLAIR hyperintensity extending beyond the CE tumor. In total, 382 patients with newly diagnosed *IDH*-wildtype glioblastoma were screened; 105 fulfilled the imaging and Ki-67 inclusion criteria. Of these, 17 were excluded because of severe artifacts or corrupted/incomplete DSC data, and 7 because no T2/FLAIR hyperintensity extended beyond the CE tumor, resulting in a final study cohort of 80 patients. All demographics and clinical covariates were extracted from clinical records.

### MRI Acquisition

Clinical MRI was performed on 1.5T (Magnetom Sola Fit, Siemens Healthineers, Erlangen, Germany) and 3T scanners (Signa HD, GE Healthcare, Illinois, USA; Magnetom Vida, Siemens Healthineers). GRE-DSC perfusion used gadobutrol with a standardized dosing scheme consisting of a preload dose (0.05 mmol/kg) followed by a DSC bolus (0.1 mmol/kg at 3-5 mL/s) and a 20 mL saline flush; the preload-to-DSC delay was approximately 5-6 minutes. Because imaging was acquired under routine clinical scanner protocols, GRE-DSC sequence parameters (TR/TE, flip angle, temporal resolution) were not prospectively standardized across scanners; scanner‑specific acquisition parameters are provided in Supplementary Table S1.

### Image Post-Processing and Physiologic Parameter Maps

GRE-DSC perfusion data were post-processed with a vendor-agnostic pipeline (Neurosuite v16.1, Cercare Medical, Aarhus, Denmark) including automated arterial input function estimation, deconvolution-based residue function estimation, and leakage and motion correction. This yielded model-based estimates of CBV as well as capillary function and oxygenation metrics (CTH, OEF, and CMRO_2_). The CTH reflects the dispersion (standard deviation) of the fitted capillary transit‑time distribution.^12^^,^^13^ The OEF is computed by integrating a single‑capillary extraction model over the fitted capillary transit‑time distribution.^10^^,^^11^^,^^16^ A relative CMRO_2_ index is derived from perfusion and extraction (CMRO_2_ ∝ CBF×OEF, assuming constant arterial oxygen content).^10^ All parameters were treated as model‑specific MRI‑based relative indices of microvascular volume and oxygen handling rather than as absolute PET-equivalent values.

### Region of Interest Definition

Region of interests (ROI) were placed on co-registered structural images by a board-certified neuroradiologist, blinded to perfusion and diffusion parametric maps. Tissue classes were defined as: (i) non-CE, T2/FLAIR-hyperintense PBZ. PBZ ROIs were restricted to within ≈20 mm of the outer CE rim to standardize sampling to an operational window commonly used in peritumoral ring approaches and comparable to the centimeter-scale region where local progression is most frequent.^17^^,^^18^ (ii) CE tumor core on post-contrast T1-weighted imaging, and (iii) contralateral NAWM without T2/FLAIR abnormality. For each tissue class, up to three non-overlapping circular 2D ROIs (≈50 mm^2^) were placed in spatially distinct locations within the tissue class, avoiding macroscopic necrosis/hemorrhage, cortical gray matter, visible vessels, cerebrospinal fluid, and susceptibility or motion artifacts. NAWM ROIs were preferentially placed in deep white matter at similar slice levels. Two examples of ROI placement are shown in Supplementary Figures S1 and S2. Parametric maps were co-registered to T2/FLAIR and post-contrast T1-weighted images prior to extraction of mean voxel values.

### Harmonization and Normalization

To mitigate scanner and field-strength effects, we applied ComBat harmonization (empirical Bayes, location-only) to PBZ, CE, and NAWM ROI means after monotone transformations appropriate to each metric: a natural logarithm for strictly positive variables (CBV, CTH, CMRO_2_, ADC) and a logit transform for OEF.^19^^,^^20^ Batch was defined as Vendor × Field; no biological or clinical covariates were included in the ComBat model to avoid attenuating true biological effects. Harmonized values were back-transformed to native units and averaged to yield one mean per subject and tissue class. NAWM-normalized ratios were then computed for each tissue class by dividing the tissue ROI mean by the contralateral NAWM ROI mean (eg PBZ rOEF = OEF(PBZ) / OEF(NAWM); CE rOEF = OEF(CE) / OEF(NAWM)). Harmonization quality was evaluated by the proportion of NAWM variance explained by batch (ANOVA R^2^) in the transformed domain before and after ComBat for each metric.

### Pathology

All pathology data were extracted from the institutional neuropathology database. Ki-67 indices were obtained from routine immunohistochemistry and recorded as continuous percentages; reported ranges were converted to single values using midpoints. Because Ki-67 reflects routine sampling of resected tumor (typically from CE tissue) and was not spatially co-registered to ROIs, it was treated as a patient-level proliferative index in imaging-pathology association analyses. TERT mutation status and MGMT methylation were recorded when available and were analyzed exploratorily.

### Statistical Analysis

Continuous variables are reported as medians with interquartile ranges (IQR) unless stated otherwise. Perfusion and diffusion parameters were analyzed as NAWM-normalized ratios as defined above. Primary analysis was the association between PBZ metrics and Ki-67 (%). Spearman’s rank correlation coefficients (ρ) were estimated together with nonparametric 95% confidence intervals (CIs) obtained by percentile bootstrapping (4,000 resamples). Partial Spearman correlations adjusting for age and sex were computed by rank residualization (regressing ranks of the predictor and outcome on age and sex and correlating the residuals), with a two-sided permutation *P*-value based on 3,000 permutations. In addition to rank-based analyses, we fitted a linear regression model with Ki-67 (%) as the dependent variable and PBZ rOEF as the predictor, reporting the unstandardized slope (β) and coefficient of determination (R^2^); heteroscedasticity-robust (HC3) standard errors were used. In a subset with available CE ROI data (complete cases), we evaluated the associations of CE metrics with Ki-67 using the same Spearman correlations. Multiplicity was controlled using the Benjamini-Hochberg false discovery rate (BH-FDR) within the PBZ and CE metric analyses (rOEF, rCTH, rCBV, rCMRO_2_, rADC vs Ki-67).

To assess whether PBZ rOEF provides information beyond CE rOEF, we fitted multivariable linear regression models (HC3 robust standard errors) with Ki-67 as the outcome and PBZ rOEF and CE rOEF as predictors, adjusting for age and sex; we report β coefficients with 95% CIs, ΔR^2^/partial R^2^ for incremental value, and variance inflation factors (VIFs) to assess collinearity.

To estimate robustness to harmonization, we repeated the primary PBZ rOEF-Ki-67 association using non-harmonized PBZ rOEF computed from raw ROI means and compared effect estimates to the ComBat-based analysis; differences in correlation estimates were quantified by paired bootstrap estimates of Δρ with percentile 95% CIs (4,000 resamples).

To evaluate scanner/vendor effects, we estimated within-stratum Spearman correlations between PBZ rOEF and Ki-67 by field strength (3T vs 1.5T). Because vendor and field strength were partially confounded in our dataset (GE acquired at 3T only; Siemens at 1.5T and 3T), vendor effects were additionally evaluated within the 3T subset by (i) estimating within‑vendor Spearman correlations (GE 3T vs Siemens 3T) and (ii) testing potential effect modification using interaction terms in age/sex‑adjusted regression models with HC3 robust standard errors. *P*‑values from these scanner robustness analyses were interpreted as sensitivity (exploratory) assessments; when summarized, BH-FDR was applied across the set of stratified correlations and interaction tests.

All other analyses were considered exploratory. We performed a discrimination analysis for Ki-67 ≥ 10% using receiver operating characteristic (ROC) area under the curve (AUC) (and precision-recall AUC) with bootstrap 95% CIs and logistic regression with robust standard errors. Subgroup comparisons by TERT and MGMT status used Mann-Whitney U tests with Cliff’s δ and 95% CIs. Additional exploratory analyses examining rCTH-stratified rOEF-Ki-67 associations, including tertile definitions, bootstrap-based Δρ comparisons, and partial correlation analyses, are described in the Supplementary Methods. Exploratory analyses were reported without multiplicity adjustment and were considered hypothesis-generating.

Missing data were handled by pairwise deletion; no imputation was applied for molecular labels. We considered *P* < .05 (two-sided) and q < 0.10 as statistically significant for primary and secondary analyses; exploratory analyses were interpreted descriptively. All analyses were carried out in Python 3.11.8 (NumPy 1.24.0, pandas 1.5.3, SciPy 1.14.1, matplotlib 3.6.3, statsmodels 0.13.5, scitkit-learn 1.8.0, SciencePlots 2.2.0).

## Results

### Cohort and Harmonization

**Figure 1.**
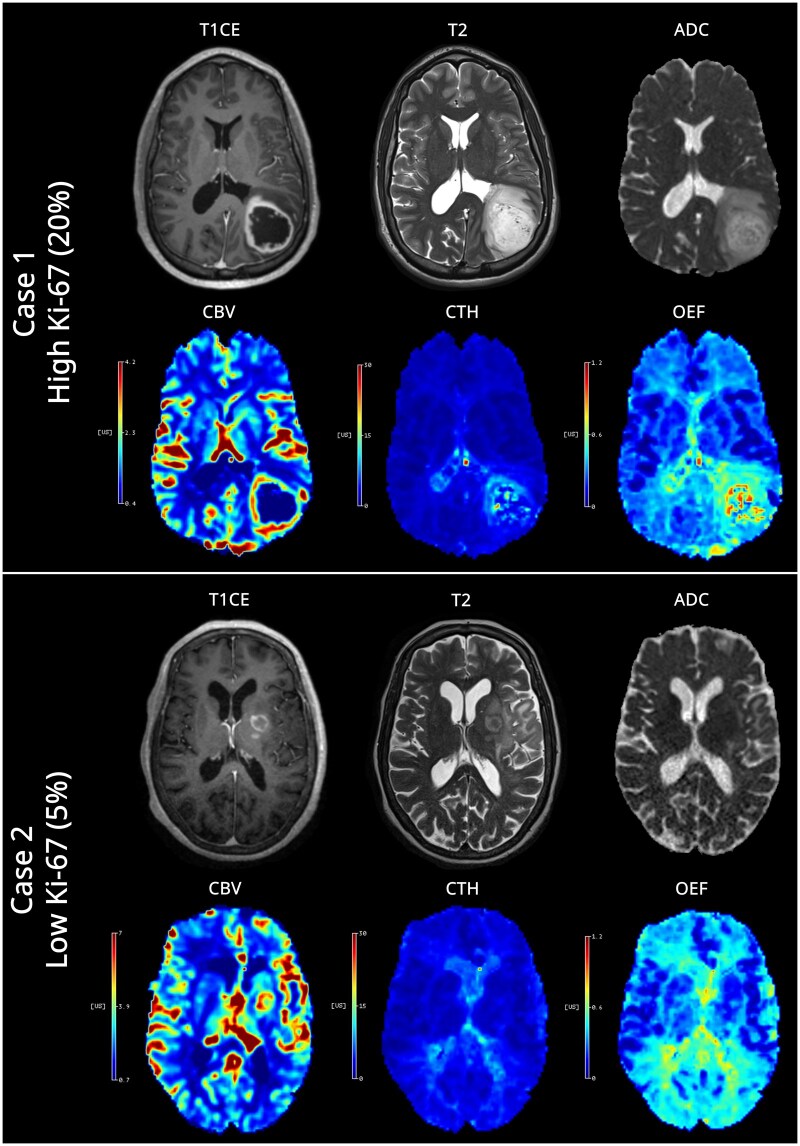
Representative multimodal MRI examples of PBZ imaging in untreated *IDH*-wildtype glioblastoma. Case 1 is a 53-year-old man with untreated *IDH*-wildtype glioblastoma (Ki-67 20%, MGMT promoter unmethylated, TERT C228T mutation) imaged on a 3T Siemens Magnetom Vida scanner. Case 2 is a 71-year-old woman with untreated *IDH*-wildtype glioblastoma (Ki-67 5%, MGMT promoter unmethylated, TERT C250T mutation) imaged on a 1.5T Siemens scanner. T1-weighted post-contrast (T1CE) images show the contrast-enhancing (CE) core. T2 turbo spin-echo (T2) images demonstrate a hyperintense peritumoral brain zone (PBZ) and contralateral normal-appearing white matter (NAWM). Apparent diffusion coefficient (ADC) maps indicate the magnitude of diffusion within tissue. Cerebral blood volume (CBV) maps highlight the hyperperfused CE core with less conspicuous PBZ signal. Capillary transit time heterogeneity (CTH) maps demonstrate heterogeneous microvascular transit in the CE core, with less pronounced PBZ abnormality in Case 1 (high Ki-67), whereas no clear PBZ CTH abnormality is apparent in Case 2 (low Ki-67). Oxygen extraction fraction (OEF) maps likewise differ between cases: Case 1 (high Ki-67) shows elevated OEF relative to NAWM in both the CE core and the PBZ, whereas no clear PBZ OEF elevation is visible in Case 2 (low Ki-67). All CBV, OEF, and CTH maps were derived from a uniform preload and leakage-corrected GRE-DSC pipeline as described in the Methods. Parametric color bars indicate relative values and are scaled separately for CBV, CTH, and OEF. Ki‑67 values shown are patient-level indices from routine pathology reporting and were not spatially co-registered to PBZ or CE ROIs.

Clinical, molecular, and baseline imaging characteristics of the cohort (*n* = 80) are summarized in Table 1, and 2 representative cases are shown in Figure 1. ComBat harmonization reduced batch-explained variance in NAWM metrics from 0.043 to 0.301 pre-ComBat to 0.000-0.018 post-ComBat across OEF, CTH, CMRO_2_, ADC, and CBV (Supplementary Table S2), consistent with mitigation of vendor and field-strength effects.

**Table 1.** Clinical, molecular, and scanner characteristics (*n* = 80)

Characteristic	Value
Age, years^a^	66.6 [56.9-76.1]
Sex (female)	37 (46.2%)
Ki-67 (%)^a^	15.0 [8.0-20.0]
*MGMT methylation status* ^b^	
not/partially methylated	52 (65%)
methylated	28 (35%)
*TERT promoter status* ^b^	
C228T mutation	46 (57.5%)
C250T mutation	21 (26.2%)
wildtype	11 (13.8%)
n.a.	2 (2.5%)
*Scanner distribution*	
Vendor (Siemens/GE)	38 (47.5%)/42 (52.5%)
Field strength (1.5T/3T)	13 (16.3%)/67 (83.7)

Abbreviations: n.a., not available; TERT, telomerase reverse transcriptase; MGMT, O^6^-methylguanine-DNA-methyltransferase.

a median [IQR].

b number (%).

### Primary Correlations of PBZ Metrics with Ki-67

**Figure 2.**
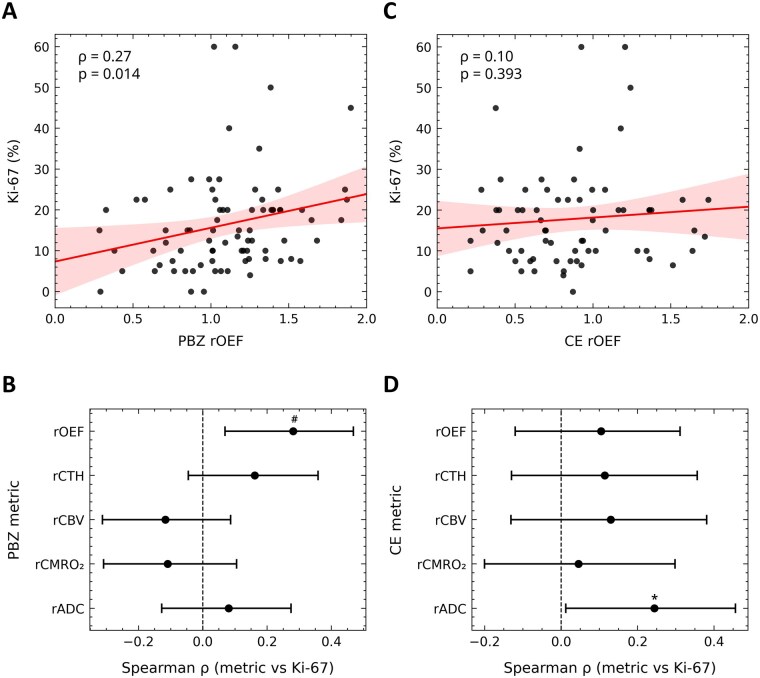
rOEF-Ki-67 association and comparison across PBZ and CE metrics. Patient-level PBZ-to-NAWM ratios were computed from ComBat-harmonized ROI means as tissue/NAWM (eg PBZ rOEF = OEF(PBZ) / OEF(NAWM)). Ki‑67 is a patient-level proliferative index obtained from routine pathology sampling and was not spatially co-registered to PBZ ROIs. (A) Scatterplot of Ki-67 (%) versus rOEF in untreated *IDH*-wildtype glioblastoma (*n* = 80). Each point represents 1 patient; PBZ rOEF was derived from ROI means averaged across the available PBZ ROIs (up to 3, depending on lesion extent) and normalized to contralateral NAWM. The solid red line shows an ordinary least-squares fit for visualization, with shaded 95% confidence band for the mean fit. PBZ rOEF was positively and significantly associated with Ki‑67. (B) Forest plot of Spearman correlations (ρ) between Ki-67 (%) and PBZ metrics (*n* = 80). Points denote Spearman’s ρ and horizontal bars show bootstrap 95% confidence intervals; the dashed vertical line marks ρ = 0. The hash (#) indicates the corresponding, solely significant BH-FDR-adjusted q-value (see Table 2). (C) Scatterplot of Ki-67 (%) versus CE rOEF (*n* = 69). Each point represents 1 patient; CE rOEF was derived from ROI means averaged across the available CE ROIs (up to 3, depending on lesion extent) and normalized to contralateral NAWM. Solid line and shaded band as in (A) are shown for visualization. CE rOEF was not associated with Ki-67. (D) Forest plot of Spearman correlations (ρ) between Ki-67 (%) and CE (*n* =69), displayed as in (B). The asterisk (*) indicates the metric with a nominal (uncorrected) *P* <.05 in this panel (see Table 4); none of the CE metrics remained significant after FDR correction. PBZ, peritumoral brain zone; NAWM, normal-appearing white matter; CE, contrast-enhancing; OEF, oxygen extraction fraction; CTH, capillary transit time heterogeneity; CBV, cerebral blood volume; CMRO_2_, cerebral metabolic rate of oxygen; ADC, apparent diffusion coefficient; BH-FDR, Benjamini-Hochberg false discovery rate.

Across the PBZ (*n* = 80), rOEF was positively associated with Ki-67 (Spearman ρ = 0.27, 95% CI 0.06-0.47; *P* = .014; q = 0.06; Figure 2A; Table 2). This association remained similar after adjustment for age and sex (partial Spearman ρ = 0.28; permutation *P* = .008). In a corroborative robust linear regression (PBZ-only model), higher rOEF was associated with higher Ki-67, consistent with the rank-based analyses. Full model coefficients (reported per 0.1-unit rOEF), confidence intervals, and fit statistics are provided in Table 3.

**Table 2.** Associations between PBZ metrics and Ki-67 (%) in untreated *IDH*-wildtype glioblastoma (*n* = 80)

PBZ metric-Ki-67 correlations	Spearman ρ	95% CI^a^	*P* ^b^	FDR q^c^
rOEF	**0.27**	**0.06-0.46**	**.014**	**0.06**
rCTH	0.16	−0.05-0.36	.153	0.38
rCBV	−0.12	−0.31-0.09	.303	0.42
rCMRO₂	−0.11	−0.31-0.10	.335	0.42
rADC	0.08	−0.13-0.27	.477	0.48

Bold values indicate nominally significant p-values (*p* < .05) and Spearman ρ values with an absolute magnitude > 0.20. FDR q-values are provided for multiple-comparison adjustment.

Abbreviations: PBZ, peritumoral brain zone; NAWM, normal-appearing white matter, CBV, cerebral blood volume; CTH, capillary transit-time heterogeneity; OEF, oxygen extraction fraction; CMRO_2_, cerebral metabolic rate of oxygen; ADC, apparent diffusion coefficient.

a Spearman ρ confidence intervals (CIs) are percentile bootstrap 95% Cis (4,000 resamples).

b *P*-values are analytic Spearman *P*-value.

c FDR q-values use Benjamini-Hochberg correction across the 5 PBZ metrics (rOEF, rCTH, rCBV, rCMRO_2_, rADC).

Among the other PBZ metrics, associations with Ki-67 were weak and not statistically significant after BH-FDR correction (Figure 2B, Table 2). Specifically, rCTH showed a small positive correlation (ρ = 0.16; *P* = .153; q = 0.38), whereas rCBV(ρ = −0.12; *P* = .303; q = 0.42), rCMRO_2_ (ρ = −0.11; *P* = .335; q = 0.42), and rADC (ρ = 0.08; *P* = .477; q = 0.48) were small with confidence intervals spanning zero. Within the PBZ cohort, rCTH and rOEF were strongly positively correlated (Spearman ρ = 0.75; *P* < .001; Figure 2B), indicating close coupling of these indices within the capillary-function framework.

### Secondary Correlations of CE Metrics with Ki-67 and Incremental Value

CE ROI data were available in 69/80 patients. CE rOEF was not associated with Ki-67 (ρ = 0.10, 95% CI −0.12-0.32; *P* = .393; q = 0.49; Table 4). Across the 5 CE metrics, none were significant after BH-FDR correction (Table 4); the largest CE correlation was observed for rADC (ρ = 0.24, 95% CI 0.01-0.46; *P* = .043; q = 0.22). CE rOEF was not associated with Ki‑67 in the CE-only regression model (CE-base model; *n*  =  69). When both CE rOEF and PBZ rOEF were included (CE+PBZ incremental model; *n* = 69), PBZ rOEF remained independently associated with Ki‑67 and improved model fit (ΔR^2^ = 0.094) with minimal collinearity (VIF ≈ 1). Full model coefficients (reported per 0.1-unit rOEF), confidence intervals, and fit statistics are provided in Table 3.

**Table 3.** Robust linear regression models of Ki-67 using NAWM-normalized rOEF ratios

Parameter	PBZ-only model	CE-base model	CE+PBZ incremental model
rOEF(CE), β per 0.1-unit	—	2.01	1.74
95% CI	—	−0.84-4.86	−1.43-4.92
p	—	0.164	0.277
rOEF(PBZ), β per 0.1-unit	0.83	—	**2.01**
95% CI	0.21 − 1.45	—	**0.08-3.93**
p	0.009	—	**0.041**
Age, β per year	—	0.06	0.13
95% CI	—	−0.37-0.48	−0.27-0.52
p	—	0.797	0.526
Sex (female), β	—	−3.18	−1.95
95% CI	—	−11.54-5.18	−10.16-6.25
p	—	0.449	0.639
n	80	69	69
R²	0.066	0.069	0.163
ΔR² vs CE-only model	—	—	**0.094**
Partial R² for PBZ block	—	—	**0.101**
VIF range	—	1.01-1.06	1.01-1.06

In a sensitivity model additionally adjusting for batch (vendor×field) in the CE-available subset, the PBZ rOEF coefficient remained similar (β  =  1.97 per 0.1-unit, 95%, CI 0.04 − 3.89, *P* = .045); CE rOEF was β  =  1.86 per 0.1-unit (95%, CI −1.36-5.07, *P* = .257). ΔR^2^ remained 0.094 and partial R^2^ for the PBZ block was 0.102. Collinearity was minimal (VIFs: CE rOEF 1.02, PBZ rOEF 1.04, age 1.06, sex 1.01). HC3 robust standard errors; PBZ-only model uses PBZ rOEF as the sole predictor in the full cohort (Ki 67 ∼ rOEF(PBZ); *n* = 80). CE-base und CE+PBZ incremental models are adjusted for age and sex in the CE-available subset (Ki 67 ∼ rOEF(CE) + age + sex; Ki 67 ∼ rOEF(CE) + rOEF(PBZ) + age + sex; *n* = 69). Primary models use ComBat-harmonized ratios; rOEF predictors are scaled per 0.1 unit for presentation. Bold type indicates reader-oriented emphasis of the prespecified key PBZ-related estimates and incremental model-fit indices; it does not denote statistical significance.

Abbreviations: CE, contrast-enhancing tumor core; PBZ, peritumoral brain zone; NAWM, normal-appearing white matter; OEF, oxygen extraction fraction; Ki‑67, Ki‑67 labeling index (%); β, unstandardized regression coefficient (reported per 0.1-unit increase in rOEF); CI, confidence interval; HC3, heteroscedasticity-consistent (HC3) robust standard errors; R^2^, coefficient of determination; ΔR^2^, change in R^2^ between nested models; partial R^2^, partial coefficient of determination for the PBZ block (incremental contribution of PBZ rOEF); VIF, variance inflation factor; *n*, sample size; vendor × field (batch), scanner vendor-by-field-strength stratum used for batch adjustment in sensitivity models.

**Table 4.** Associations between CE metrics and Ki-67 (%) in untreated *IDH*-wildtype glioblastoma (*n* = 69)

CE r-metric-Ki-67 correlations	Spearman ρ	95% CI^a^	*P* ^b^	FDR q^c^
rOEF	0.10	−0.12-0.32	0.393	0.49
rCTH	0.11	−0.13-0.36	0.352	0.49
rCBV	0.13	−0.12-0.38	0.288	0.49
rCMRO₂	0.05	−0.20-0.30	0.712	0.71
rADC	0.24	0.01-0.46	0.043	0.22

Abbreviations: CE, contrast-enhancing; NAWM, normal-appearing white matter, CBV, cerebral blood volume; CTH, capillary transit-time heterogeneity; OEF, oxygen extraction fraction; CMRO_2_, cerebral metabolic rate of oxygen; ADC, apparent diffusion coefficient.

a Spearman ρ confidence intervals (CIs) are percentile bootstrap 95% Cis (4,000 resamples).

b *P*-values are analytic Spearman *P*value.

c FDR q-values use Benjamini-Hochberg correction across the 5 PBZ r-metrics (rOEF, rCTH, rCBV, rCMRO_2_, rADC).

### Sensitivity and Robustness Analyses

In the no‑ComBat sensitivity analysis, the PBZ rOEF-Ki‑67 association was highly similar (ρ = 0.28, 95% CI 0.07-0.48; *P* = .011). In paired bootstrap comparison, the difference in correlation was small (Δρ = −0.001; p bootstrap = 0.365). Scanner/vendor robustness analyses suggested heterogeneity in within-stratum effect estimates. The PBZ rOEF-Ki-67 association was present in the 3T subgroup (*n* = 67; ρ = 0.31, 95% CI 0.08-0.51; *P* = .012) but was imprecise at 1.5T (*n* = 13; ρ = 0.12, 95% CI −0.57-0.72; *P* = .698). GE 3T showed a clear positive association (*n* = 42; ρ = 0.50, 95% CI 0.22-0.71; *P* < .001), whereas Siemens 3T was near null (*n* = 25; ρ = 0.05, 95% CI −0.28-0.36; *P* = .818). Formal interaction tests in age/sex-adjusted regression were not statistically supported (vendor 3T interaction β = 7.31, 95% CI −5.39-20.00; *P* = .259), so these differences are interpreted as exploratory heterogeneity rather than confirmed effect modification.

Exploratory ROC analysis of PBZ rOEF for discriminating Ki‑67 ≥ 10% showed modest performance (AUC = 0.62; Supplementary Figure S3; Supplementary Table S3) and is interpreted descriptively, as it was derived and evaluated in the same cohort. Other exploratory analyses, including rCTH-stratified evaluation of the PBZ rOEF-Ki‑67 association (Supplementary Table S4) and molecular subgroup comparisons by TERT and MGMT status (Supplementary Table S5), are provided in the Supplement and are also interpreted descriptively.

## Discussion

In this single-center MRI study of untreated *IDH*-wildtype glioblastoma, NAWM-normalized rOEF in the NCE PBZ demonstrated a modest positive association with Ki-67, whereas other PBZ metrics (rCTH, rCBV, rADC, rCMRO_2_) did not show significant associations after multiplicity control. The PBZ rOEF-Ki-67 association remained similar after adjusting for age and sex with modest effect sizes. Importantly, Ki‑67 is a proliferation marker obtained from routine pathology sampling (typically from resected CE tumor) and was not spatially co-registered to PBZ ROIs. Therefore, our findings should be interpreted as patient-level associations between PBZ oxygen-handling phenotype and global proliferative activity rather than as evidence of PBZ-local proliferation gradients or a direct imaging surrogate of infiltration.

Given the origin of Ki‑67 sampling, we performed a secondary, context-setting analysis in the CE tumor core. CE rOEF was not associated with Ki‑67 and none of the CE metrics were significant after FDR correction. In incremental regression models that included both CE and PBZ rOEF, PBZ rOEF remained associated with Ki‑67, improving explained variance with minimal collinearity, indicating that the PBZ rOEF signal is not simply a proxy for CE oxygen-handling behavior within the constraints of ROI sampling and routine pathology. Together, these results support PBZ oxygen-handling phenotyping as a physiologic dimension of non-CE peritumoral abnormality not captured by conventional structural MRI alone.

The selective association of PBZ rOEF with Ki-67 suggests that oxygen-extraction phenotyping may capture a physiologic dimension of treatment‑relevant PBZ abnormality beyond contrast enhancement while acknowledging that limited power and ROI-level tissue heterogeneity may also contribute to null findings for other metrics.^7^^,^^21^^,^^22^ Consistent with this interpretation, prior MRI studies in glioma, including analyses of CE and non-CE tumor regions, have reported associations between oxygen‑handling phenotypes, aggressive molecular features, and clinical endpoints such as overall survival and recurrence.^15^^,^^23^^,^^24^

Within flow-diffusion theory, increased transit‑time heterogeneity can reduce exchange efficiency and constrain effective oxygen extraction, providing a physiologic rationale for considering CTH as context when interpreting OEF.^10^,^11^,^25^ In our cohort, rOEF and rCTH were strongly correlated, consistent with coupling of these indices within the capillary‑function framework and reinforcing that rCTH should be interpreted primarily as context for extraction rather than as an independent proliferative marker. Prior work suggests that CTH-derived indices can add information beyond CBV for glioma grading and outcome prediction and that recurrent glioblastoma is associated with elevated CTH.^26^^,^^27^

Beyond contextualizing extraction with CTH, alternative physiologic stratification strategies could combine PBZ rOEF with vascular-capacity and oxygen-metabolism indices such as rCBV and rCMRO_2_, consistent with multiparametric glioblastoma phenotyping approaches.^21^^,^^23^^,^^24^ Such combinations require cautious interpretation because rCMRO_2_ is a model-derived perfusion-extraction composite (CMRO_2_ ∝ CBF × OEF), whereas rCBV reflects vascular volume rather than flow. Conceptually, high rOEF with high rCBV and high rCMRO_2_ could indicate increased oxygen demand with preserved metabolic throughput, whereas high rOEF with high rCBV but low or normal rCMRO_2_ could suggest flow-extraction mismatch or mixed tissue composition within ROI averages. Given the modest sample size, ROI-level averaging, and absence of spatially matched histology, we did not add post hoc rCBV/rCMRO_2_-stratified analyses; larger volumetric or habitat-based studies are better suited to test these OEF-CBV-CMRO_2_ phenotypes.

From a methodological perspective, interpreting OEF as NAWM-normalized ratio is appropriate because absolute MRI-based OEF estimates are method- and model-dependent and are not directly interchangeable with endogenous susceptibility-based approaches (eg qBOLD), which rely on different signal mechanisms and modeling assumptions.^16^ Existing work suggests reasonable agreement between MRI-based OEF estimates and ^15^O-PET at the whole-brain level.^16^^,^^28^^,^^29^ Yet, DSC-derived CTH and OEF remain model-specific indices that depend on acquisition and post-processing choices and have already shown added clinical value beyond rCBV in treatment response phenotyping.^9^^,^^14^^,^^30^ In this study, we used a uniform preload and leakage-corrected GRE-DSC pipeline and normalized PBZ ROI means to contralateral NAWM in a heterogenous DSC dataset to improve comparability across patients and scanners.^31^ ComBat harmonization substantially reduced batch-explained variance in NAWM across vendor and field strength, while the primary PBZ rOEF-Ki-67 association was robust to omission of harmonization, supporting that the main association was not driven by ComBat. Nonetheless, scanner/vendor stratified analyses showed heterogeneity in effect estimates with non-definitive interaction tests, underscoring the need for standardized protocols and cross-vendor validation before broader clinical translation.

The clinical implications of an OEF-focused PBZ signature are conceptually appealing but require cautious interpretation. The PBZ is increasingly recognized as a treatment-relevant compartment in *IDH*-wildtype glioblastoma,^3^^,^^5^ and our data suggest that higher PBZ rOEF is associated with higher Ki-67 at the patient level. At the same time, effect sizes were modest, and exploratory discrimination for Ki‑67 ≥ 10% was limited, indicating that PBZ rOEF may not be standalone classifier for proliferative status. Elevated rOEF in the NCE PBZ may reflect a stressed microvascular oxygen‑handling phenotype within a heterogeneous compartment comprising variable composition of infiltrating tumor, vasogenic edema, and reactive microenvironmental change that is highly relevant for progression after local therapy.^6^^,^^7^^,^^32^ Complementary MRI-based strategies to interrogate the treatment‑relevant PBZ, such as radiomics/machine learning-based phenotyping, connectivity-informed analyses, and microvascular perfusion approaches using spin‑echo DSC, have also been proposed but were not evaluated in this study.^33^^,^^34^ Any application to spatial targeting (eg biopsy guidance or radiotherapy margin adaptation) cannot be inferred from the current patient-level, non-co‑registered design and would require prospective studies with spatially registered sampling of non-CE PBZ tissue guided by oxygen-handling maps and outcome-based validation (eg recurrence mapping and survival endpoints) to establish incremental value over established imaging markers.

This study has several limitations. First, ROI-based sampling is inherently vulnerable to tissue heterogeneity, partial-volume, and threshold effects, which limit tissue specificity within ROI averages. Future volumetric and voxel-wise analyses will be needed to better separate infiltrative and edematous PBZ components.^21^ In addition, amino acid PET can delineate tumor tissue beyond CE, and biopsy-validation studies indicate that regions with increased FET uptake frequently contain glioma tissue or infiltration, whereas T2/FLAIR abnormality without corresponding metabolic activity may show only low-density infiltration or reactive changes.^35-37^ Future studies could therefore extract DSC-derived OEF and CTH preferentially from FET-positive non-enhancing PBZ subregions, which may increase tumor specificity and yield stronger associations with proliferative and spatially co-registered histologic markers.^35-38^ Second, ROIs were placed by a single-reader and inter-rater reproducibility was not assessed. ROI-based quantification in DSC perfusion imaging, particularly hotspot-style sampling and reference tissue placement, is known to introduce observer-dependent variability, and consensus profiles estimate that ROI placement can contribute substantial measurement variance, which is reduced when averaging multiple ROIs rather than relying on a single ROI. We attempted to mitigate observer effects by using a prespecified ROI protocol, blinding ROI placement to parametric maps, placing up to three ROIs per tissue class, and NAWM normalization. Third, pathology was not spatially co-localized to PBZ ROIs. Ki-67 reflects routine tumor sampling and cannot resolve microscopic proliferation gradients at the margin, which likely dilutes spatial specificity.^4^^,^^39^ Fourth, effect sizes for PBZ imaging-proliferation links were modest, and the cohort size and missing molecular labels limited power for exploratory molecular subgroup comparisons. Fifth, generalizability may be limited by acquisition and implementation factors. GRE-DSC sequence parameters were dictated by routine clinical scanner protocols and were therefore not prospectively standardized to a single consensus-recommended acquisition, and DSC-derived OEF/CTH remain model- and implementation-dependent indices. Although we mitigated scanner-related effects using leakage correction, NAWM normalization, and ComBat harmonization, effect estimates showed vendor/field heterogeneity, underscoring the need for prospective multicenter validation under consensus-aligned acquisition and processing before clinical utility can be established.^8^^,^^9^ Moreover, DSC-derived OEF/CTH estimation is currently platform- and implementation-dependent, which limits immediate reproducibility across post-processing environments. Finally, this was a single-center baseline study without survival endpoints; generalizability and clinical utility should be tested prospectively across centers and vendors under standardized protocols with spatially matched tissue sampling outcome data.

In summary, PBZ rOEF showed a modest positive association with Ki-67 in untreated *IDH*-wildtype glioblastoma, whereas other PBZ metrics and all CE metrics did not show significant associations after multiplicity control. These findings support NAWM-normalized PBZ oxygen-handling phenotyping as a candidate imaging marker associated with global proliferative activity at the patient level and motivate prospective multicenter validation with standardized protocols, spatially matched tissue sampling, and outcome endpoints to define reproducibility and clinical relevance.

## Supplementary Material

vdag163_Supplementary_Data

## Data Availability

Data reported in this article can be shared in compliance with current data protection regulations by the European Union and approval from the relevant regulatory authorities. All proposals should be directed to the corresponding author.
