# Supplementary material for: Peritumoral brain zone oxygen extraction fraction is associated with tumor Ki-67 index in untreated glioblastoma
Source: Neurooncol Adv. 2026 Jun 18;8(1):vdag163. doi: 10.1093/noajnl/vdag163 (PMC13348716; doi:10.1093/noajnl/vdag163)
Supplement: vdag163_Supplementary_Data [file vdag163_supplementary_data.docx]

**Supplementary Methods**

*Partial Spearman correlations*

Partial Spearman correlations adjusting for age and sex were implemented by a rank-residualization approach. For a given predictor X and outcome Y, we first replaced X and Y by their ranks. We then regressed each set of ranks on the covariates (age, sex) using ordinary least squares and computed the Pearson correlation between the resulting residuals. To obtain a nonparametric p-value, we permuted the residualized ranks of Y (under the null of no association), recomputed the partial correlation for each permutation and calculated a two-sided permutation p-value based on 3,000 permutations.

*CTH tertiles*

To examine whether the strength of the rOEF–Ki‑67 association varied across microvascular transit‑heterogeneity states, we defined rCTH tertiles on the distribution of rCTH in the PBZ cohort and re-estimated the rOEF–Ki-67 correlation within each tertile. rCTH tertiles were defined once based on the distribution of rCTH. Each tertile (Low, Mid, High) contained approximately one third of PBZ cases. For each tertile, we report Spearman ρ (with bootstrap 95% CI) between rOEF and Ki-67; the High-Low Δρ contrast is summarized by its bootstrap CI and p-value as described above. Differences in correlation between tertiles (e.g., High vs Low) were quantified using bootstrapped estimates of Δρ with percentile 95% CIs and two-sided bootstrap p-values (4,000 resamples). For differences in correlation between strata (Δρ in high versus low rCTH tertiles), we resampled each stratum independently with a replacement and re-estimated ρ in each stratum. Then we computed Δρ for that bootstrap replicate, and repeated this 4,000 times. The empirical distribution of Δρ across bootstraps was used to obtain a 95% CI and a two-sided bootstrap p-value.

**Supplementary Tables**

**Supplementary Table S1. GRE-DSC perfusion acquisition parameters**

| **Scanner** | **TR (ms)** | **TE (ms)** | **Flip angle (°)** | **Temporal resolution (s)** |
| --- | --- | --- | --- | --- |
| GE Signa HD | 1400 | 20 | 60 | 1.40 |
| Siemens Magnetom Vida | 1600 | 30 | 90 | 1.60 |
| Siemens Magnetom Sola fit | 2770 | 31 | 90 | 2.77 |

Flip angle, temporal resolution, and TR/TE settings reflect each vendor’s recommended tumor-perfusion protocol for the specified field strength.

Abbreviations: TR, repetition time; TE, echo time; GRE-DSC, gradient-echo dynamic susceptibility contrast.

**Supplementary Table S2. NAWM batch variance (R²) before and after location-only ComBat harmonization for PBZ metrics**

| **Metric** | **Batch R² pre-ComBat^a^** | **Batch R² post-ComBat^a^** |
| --- | --- | --- |
| NAWM OEF | 0.043 | 0.000 |
| NAWM CTH | 0.261 | 0.000 |
| NAWM CMRO2 | 0.172 | 0.018 |
| NAWM ADC | 0.243 | 0.000 |
| NAWM CBV | 0.301 | 0.001 |

Abbreviations: NAWM, normal-appearing white matter; PBZ, peritumoral brain zone; CBV, cerebral blood volume; CTH, capillary transit-time heterogeneity; OEF, oxygen extraction fraction; CMRO₂, cerebral metabolic rate of oxygen.

^a^ Batch R² reflects the proportion of NAWM variance explained by batch (Vendor × Field) in a one-way ANOVA of the transformed domain before and after location-only ComBat harmonization.

**Supplementary Table 3. Exploratory discrimination performance of PBZ rOEF**.

| **Parameter** | **Result** |
| --- | --- |
| Outcome definition | Ki-67 ≥ 10% (midpoint-converted where ranges were reported) |
| Predictor | PBZ rOEF = ratio_PBZ_OEF_to_NAWM_ComBat |
| Sample size | 80 |
| Event prevalence | 72.5% |
| ROC AUC | 0.62 (95% CI 0.48–0.76) |
| PR-AUC | 0.81 (95% CI 0.69–0.91) |
| Chosen cutpoint | rOEF ≥ 0.98 (Youden J, same-sample estimate) |
| Sensitivity at cutpoint | 0.78 (95% CI 0.68–0.88) |
| Specificity at cutpoint | 0.50 (95% CI 0.29–0.71) |
| Logistic OR per 0.1-unit rOEF | 1.12 (95% CI 0.98–1.29); p = 0.107 |
| Logistic OR per 1.0-unit rOEF | 3.11 (95% CI 0.78–12.40) |
| Model fit | McFadden pseudo-R² = 0.028 |

AUC, PR-AUC, sensitivity, and specificity confidence intervals are percentile bootstrap 95% confidence intervals based on 4,000 resamples. The cutpoint was selected by Youden J in the same dataset and therefore represents an optimistic, exploratory same-sample estimate. PR-AUC should be interpreted in the context of the high event prevalence. Logistic odds ratios were estimated using a binomial model with HC3 robust standard errors.

Abbreviations: AUC, area under the receiver operating characteristic curve; PR-AUC, area under the precision-recall curve; PBZ, peritumoral brain zone; rOEF, NAWM-normalized oxygen extraction fraction.

**Supplementary Table S4. Exploratory PBZ rCTH tertile stratification of the PBZ rOEF**–**Ki-67 association (n = 80)**

| **rCTH tertile** | **Definition^a^** | **n** | **rOEF^b^** | **Spearman ρ (95% CI)^c^** | **p (Spearman)** |
| --- | --- | --- | --- | --- | --- |
| Low | rCTH ≤ 0.81 | 27 | 0.83 [0.60–1.01] | -0.15 (-0.24–0.48) | 0.461 |
| Mid | 0.81 < rCTH ≤ 1.29 | 26 | 1.10 [1.01–1.32] | 0.29 (0.09–0.60) | 0.153 |
| High | rCTH > 1.29 | 27 | 1.33 [1.23–1.49] | 0.29 (-0.11–0.63) | 0.141 |

| **Contrast** | **Δρ^d^** | **95% CI** | **p (bootstrap)** |
| --- | --- | --- | --- |
| High–Low rCTH | 0.14 | -0.41–0.64 | 0.635 |

Abbreviations: CTH, capillary transit-time heterogeneity; OEF, oxygen extraction fraction.

^a^ Tertile cut-points were defined once from the full PBZ cohort distribution of rCTH (q1 = 0.81, q2 = 1.29).

^b^ median [IQR].

^c^ Within-tertile Spearman ρ CIs use bootstrap resampling (4,000 resamples).

^d^ Δρ uses independent within-tertile bootstraps (4,000 resamples) and a two-sided tail-area bootstrap p-value.

**Supplementary Table S5. Associations between PBZ imaging metrics and TERT mutation (C228T/C250T) or MGMT methylation status**

| **Metric^a^** | **TERT-mutated^b^ (n=67)** | **TERT-wildtype^b^ (n=11)** | **Mann-Whitney p** | **Cliff's δ** |
| --- | --- | --- | --- | --- |
| rOEF | 1.11 [0.87–1.33] | 1.12 [1.04–1.52] | 0.189 | -0.25 |
| rCTH | 0.97 [0.70–1.34] | 1.28 [1.01–1.82] | 0.022 | -0.43 |
| rCBV | 1.98 [0.99–3.25] | 1.59 [1.31–2.58] | 0.482 | 0.13 |
| rADC | 1.35 [1.15–1.84] | 1.55 [1.13–1.76] | 0.966 | -0.01 |
| rCMRO₂ | 1.85 [0.99–3.00] | 1.51 [1.20–2.30] | 0.473 | 0.14 |
| **Metric^a^** | **MGMT m^b^ (n=28)** | **MGMT pm/nm^b^ (n=52)** | **Mann-Whitney p** | **Cliff's δ** |
| rOEF | 1.07 [0.73–1.30] | 1.10 [0.98–1.36] | 0.285 | -0.15 |
| rCTH | 0.89 [0.64–1.43] | 1.09 [0.78–1.45] | 0.209 | -0.17 |
| rCBV | 2.16 [1.11–3.25] | 1.80 [0.90–3.21] | 0.928 | 0.01 |
| rADC | 1.40 [1.24–2.07] | 1.34 [1.13–1.77] | 0.124 | 0.21 |
| rCMRO₂ | 1.86 [1.13–2.82] | 1.78 [0.82–2.80] | 0.766 | 0.04 |

Abbreviations: PBZ, peritumoral brain zone; NAWM, normal-appearing white matter; CBV, cerebral blood volume; CTH, capillary transit-time heterogeneity; OEF, oxygen extraction fraction; CMRO₂, cerebral metabolic rate of oxygen; ADC, apparent diffusion coefficient; TERT, telomerase reverse transcriptase; MGMT, O⁶-methylguanine-DNA-methyltransferase; m, methylated; pm, partially methylated; nm, not methylated.

**Supplementary Figures**


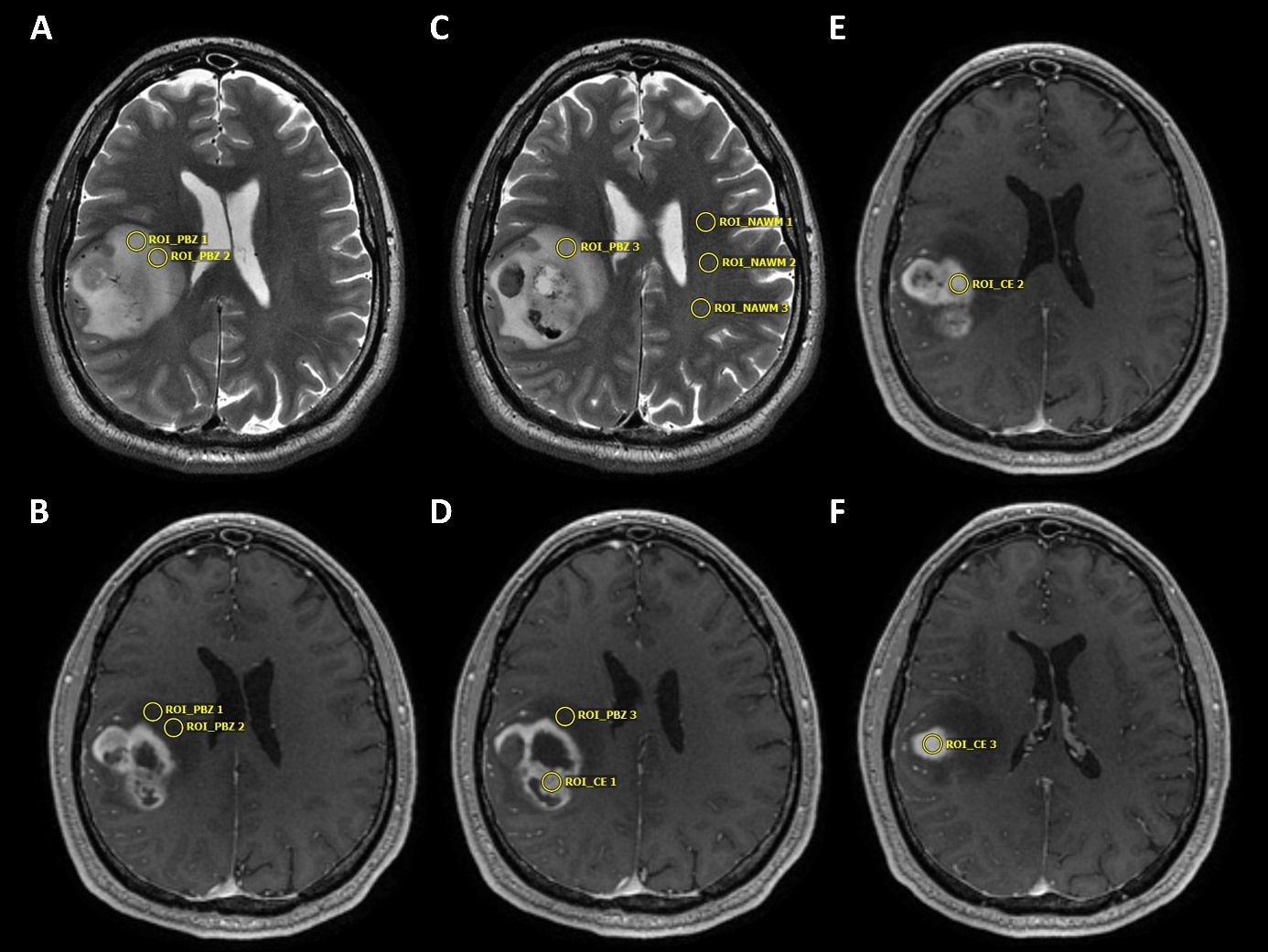


**Supplementary Figure S1. Representative ROI placement examples for PBZ, NAWM, and CE compartments (Example 1).**

A board-certified neuroradiologist placed circular 2D ROIs (area = 49.5 mm^2^; diameter = 7.9 mm) on co-registered structural images while blinded to perfusion and diffusion parametric maps; parametric maps were co-registered to the same structural images for ROI extraction. PBZ ROIs were placed within non–contrast-enhancing peri-enhancing T2/FLAIR hyperintensity and restricted to within ≈20 mm of the outer contrast-enhancing rim on co-registered T1CE; NAWM ROIs were placed in contralateral normal-appearing white matter without T2/FLAIR abnormality; CE ROIs were placed within contrast-enhancing tumor core on T1CE. Up to three ROIs per tissue class were placed in spatially distinct locations. **(A)** T2: PBZ ROI within peri-enhancing T2 hyperintensity. **(B)** T1CE: same PBZ ROI projected onto the co-registered T1CE, demonstrating location outside the enhancing core and within ≈20 mm of the enhancing rim. **(C)** T2: PBZ ROI within T2/FLAIR abnormality and a contralateral NAWM ROI at a comparable slice level (non-overlapping). **(D)** T1CE: PBZ ROI projection outside enhancement (within ≈20 mm of the rim) and a CE ROI placed within enhancing tumor core. **(E–F)** T1CE: additional CE ROI placements (examples) within enhancing tumor core (up to three when feasible).

**Abbreviations:** ROI, region of interest; PBZ, peritumoral brain zone; NAWM, normal-appearing white matter; CE, contrast-enhancing tumor core; T1CE, post-contrast T1-weighted imaging.

**Alt text:** Structural MRI panels illustrate representative manual circular ROI placement in the PBZ, contralateral NAWM, and contrast-enhancing tumor core, with PBZ ROIs positioned outside enhancement and within approximately 20 mm of the enhancing rim.


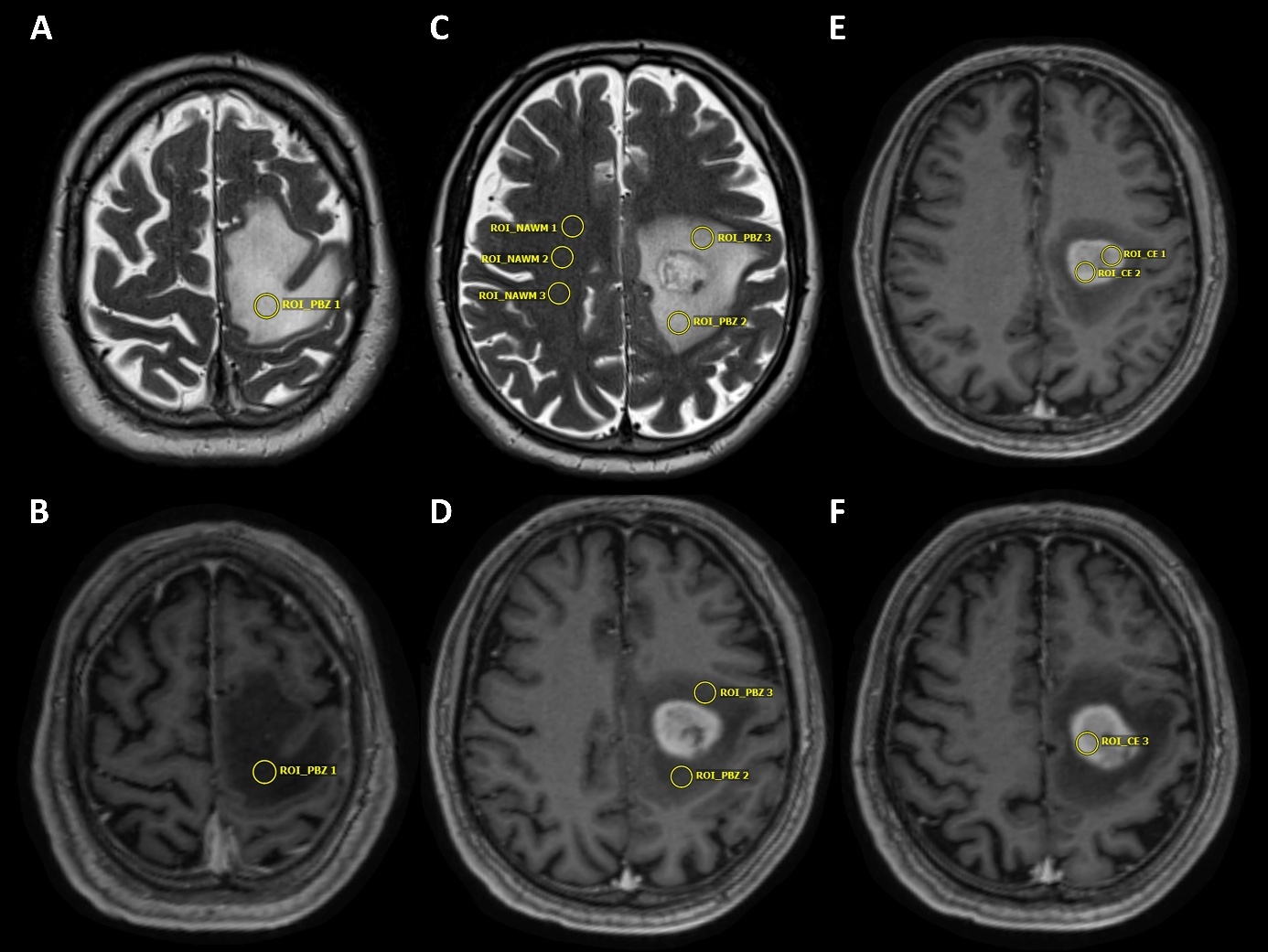


**Supplementary Figure S2. Representative ROI placement examples for PBZ, NAWM, and CE compartments (Example 2).**

ROIs placement (area = 51.8 mm^2^; diameter = 8.1 mm) was performed identical as described in Supplementary Figure S1. **(A)** T2: PBZ ROI within peri-enhancing T2 hyperintensity. **(B)** T1CE: same PBZ ROI projected onto co-registered T1CE, confirming non–contrast enhancement and ≤≈20 mm distance from the enhancing rim. **(C)** T2: multiple PBZ ROIs within T2 abnormality and a contralateral NAWM ROI at a comparable slice level (non-overlapping). **(D)** T1CE: PBZ ROIs projected onto co-registered T1CE, demonstrating location outside enhancement and within ≈20 mm of the enhancing rim. **(E–F)** T1CE: CE ROI placements within enhancing tumor.

**Abbreviations:** ROI, region of interest; PBZ, peritumoral brain zone; NAWM, normal-appearing white matter; CE, contrast-enhancing tumor core; T1CE, post-contrast T1-weighted imaging.

**Alt text:** Structural MRI panels illustrate representative manual circular ROI placement in the PBZ, contralateral NAWM, and contrast-enhancing tumor core, with PBZ ROIs positioned outside enhancement and within approximately 20 mm of the enhancing rim.


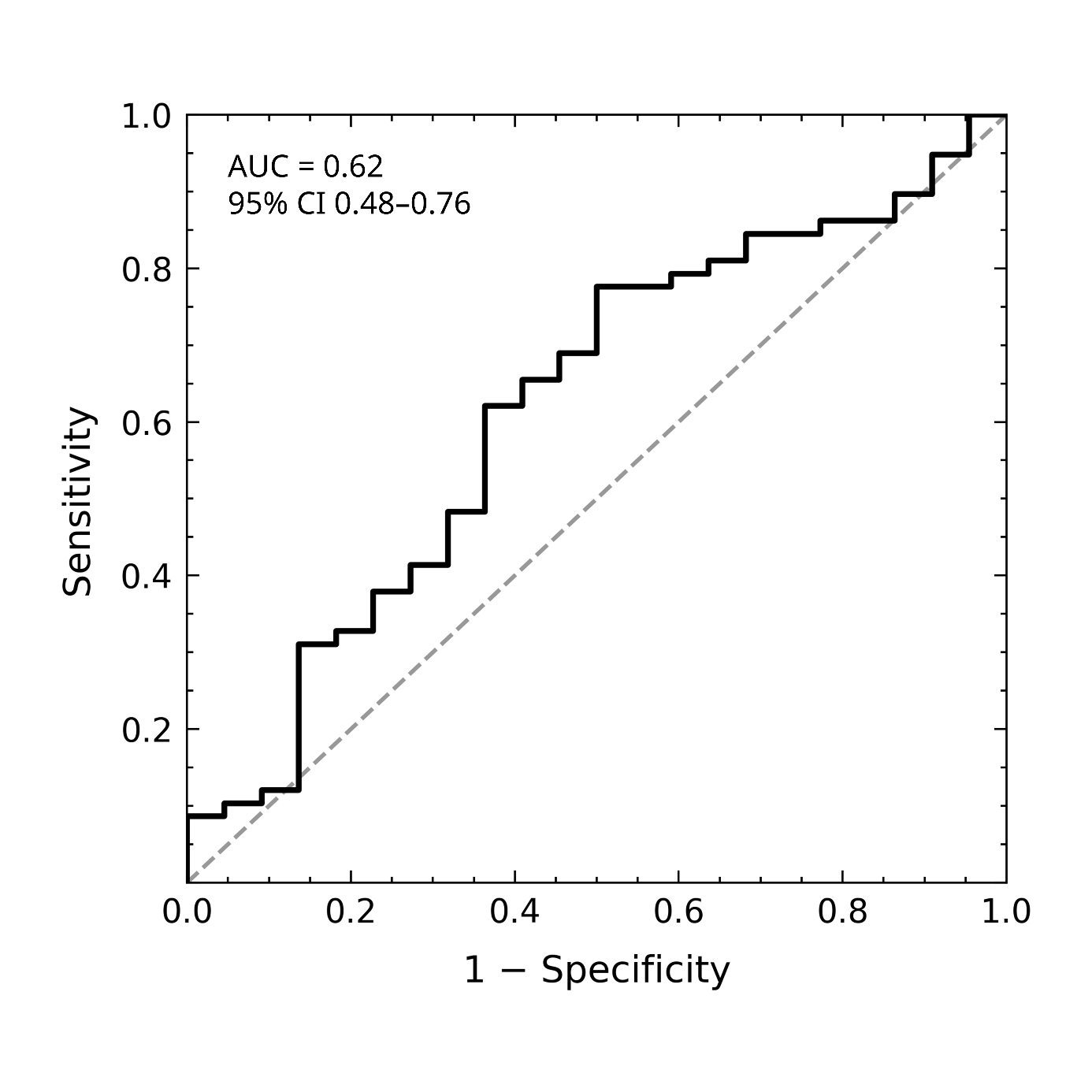
**Supplementary Figure S3. Exploratory receiver operating characteristic (ROC) analysis of PBZ rOEF for discrimination of tumors with Ki-67 ≥10% versus Ki-67 <10%.**

The predictor was the NAWM-normalized PBZ OEF ratio. The ROC curve is shown in black, with the diagonal dashed line indicating no discriminatory performance. Discriminatory performance was modest. Because both the Youden cutpoint and performance estimates were derived in the same cohort, this analysis should be interpreted as exploratory.

**Abbreviations:** ROC, receiver operating characteristic; PBZ, peritumoral brain zone; rOEF, NAWM-normalized oxygen extraction fraction; NAWM, normal-appearing white matter; AUC, area under the receiver operating characteristic curve.

**Alt text:** ROC curve for PBZ rOEF discrimination of Ki-67 ≥10% shows modest performance, with the curve only slightly above the diagonal no-discrimination line.
